# *catena*-Poly[[[tetra­aqua­nickel(II)]-μ-4,4′-bipyridyl-κ^2^ *N*:*N*′] 3,3′-(*p*-phenyl­ene)diacrylate]

**DOI:** 10.1107/S1600536811036993

**Published:** 2011-09-17

**Authors:** Ni-Ya Li

**Affiliations:** a College of Chemistry and Materials Science, Huaibei Normal University, Huaibei 235000, Anhui, People’s Republic of China

## Abstract

In the title compound, {[Ni(C_10_H_8_N_2_)(H_2_O)_4_](C_12_H_8_O_4_)}_*n*_, the Ni^II^, 4,4′-bipyridyl (bipy) and 3,3′-(*p*-phenyl­ene)diacrylate (*L* ^2−^) moieties are situated on inversion centres. The bipy ligands bridge Ni^II^ ions into positively charged polymeric chains along [101]. The Ni^II^ atom is coordinated by two N atoms from two bipy ligands and four water mol­ecules in a distorted octa­hedral geometry. *L* ^2−^ anions inter­act with the polymeric chains *via* O–H⋯O hydrogen bonds, forming a three-dimensional supra­molecular network.

## Related literature

For a metal-organic complex with bipy and *L* ^2−^ ligands, see: Huang *et al.* (2008 ▶). For related Ni complexes, see: Batten & Harris (2001 ▶); Dong (2009 ▶); Li *et al.* (2010 ▶).
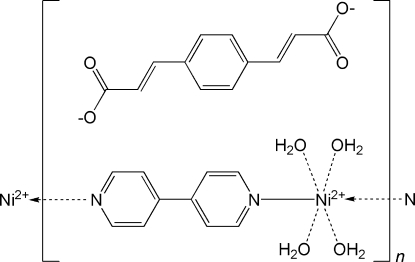


## Experimental

### 

#### Crystal data

[Ni(C_10_H_8_N_2_)(H_2_O)_4_](C_12_H_8_O_4_)
*M* *_r_* = 503.12
Triclinic, 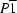

*a* = 7.0867 (14) Å
*b* = 7.3614 (15) Å
*c* = 10.418 (2) Å
α = 95.51 (3)°
β = 102.51 (3)°
γ = 97.27 (3)°
*V* = 522.0 (2) Å^3^
*Z* = 1
Mo *K*α radiation
μ = 0.98 mm^−1^
*T* = 223 K
0.40 × 0.40 × 0.25 mm

#### Data collection

Rigaku Mercury CCD area-detector diffractometer
Absorption correction: multi-scan (*REQAB*; Jacobson, 1998 ▶) *T*_min_ = 0.694, *T*_max_ = 0.791
4910 measured reflections
1884 independent reflections
1807 reflections with *I* > 2σ(*I*)
*R*_int_ = 0.018

#### Refinement

*R*[*F* ^2^ > 2σ(*F* ^2^)] = 0.026
*wR*(*F* ^2^) = 0.067
*S* = 1.07
1884 reflections
167 parameters
H atoms treated by a mixture of independent and constrained refinement
Δρ_max_ = 0.41 e Å^−3^
Δρ_min_ = −0.36 e Å^−3^

### 

Data collection: *CrystalClear* (Rigaku, 2001 ▶); cell refinement: *CrystalClear*; data reduction: *CrystalStructure* (Rigaku/MSC, 2004 ▶); program(s) used to solve structure: *SHELXTL* (Sheldrick, 2008 ▶); program(s) used to refine structure: *SHELXTL*; molecular graphics: *SHELXTL*; software used to prepare material for publication: *SHELXTL* and *PLATON* (Spek, 2009 ▶).

## Supplementary Material

Crystal structure: contains datablock(s) I, global. DOI: 10.1107/S1600536811036993/cv5150sup1.cif

Structure factors: contains datablock(s) I. DOI: 10.1107/S1600536811036993/cv5150Isup2.hkl

Additional supplementary materials:  crystallographic information; 3D view; checkCIF report

## Figures and Tables

**Table 1:** Hydrogen-bond geometry (Å, °)

*D*—H⋯*A*	*D*—H	H⋯*A*	*D*⋯*A*	*D*—H⋯*A*
O1—H2*W*⋯O3	0.84 (3)	1.90 (3)	2.734 (2)	170 (3)
O1—H1*W*⋯O3^i^	0.79 (3)	1.90 (3)	2.683 (2)	171 (3)
O2—H3*W*⋯O4^ii^	0.85 (3)	1.86 (3)	2.701 (2)	172 (3)
O2—H4*W*⋯O4^iii^	0.82 (3)	1.95 (3)	2.754 (2)	167 (3)

Symmetry codes: (i) 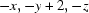; (ii) 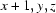; (iii) 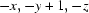.

**Fig. 1.**
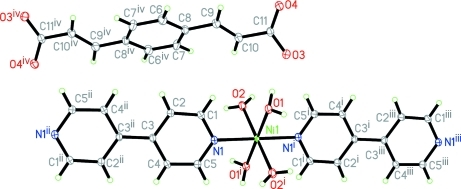
A portion of the crystal structure of (I) showing the atomic numbering and 30% probability displacement ellipsoids [symmetry codes: (i) -x + 1, -y + 2, -z; (ii) -x + 2, -y + 2, -z + 1; (iii) x - 1, y, z - 1; (iv) -x + 1, -y + 1, -z + 1].

**Fig. 2.**
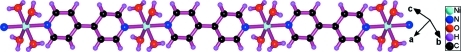
View of the positively charged polymeric chain in (I).

**Fig. 3.**
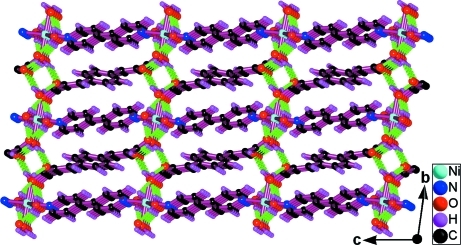
View of the three-dimensional supramolecular network of the title compound. The green dashed lines represent intermolecular hydrogen bonds.

## supplementary crystallographic information

### Comment

In recent years, supramolecular frameworks have attracted considerable attention because of their intriguing architectures and potential applications (Li *et al.*, 2010). Polycarboxylates and dipyridyl ligands have proved to be good linkers for the construction of supramolecular compounds (Li *et al.*, 2010). In this paper, we report the hydrothermal synthesis and structure of a supramolecular compound assembled by the mixed ligands of 4,4'-bipyridyl (bipy) and 3,3'-(1,4-phenylene)-diacrylate (*L*^2-^), respectively.

The aymmetric unit of the title compound (I) (Fig. 1) contains half of a [Ni(H_2_O)_4_(bipy)] unit, half of a *L*^2-^ anion (*L*^2-^ = 3,3'-(1,4-phenylene)-diacrylate) and two water molecules. Each Ni center has a distorted octahedral environment being coordinated by four water molecules at the basal positions and two N atoms from two different bipy ligand at the apical position. The Ni–O and Ni–N bond lengths are comparable with those in reported Ni-complexes (Batten & Harris, 2001; Dong, 2009; Li *et al.*, 2010). The Ni centers are bridged by bipy ligands to form one-dimensional [Ni(H_2_O)_4_(bipy)]_n_ polymeric chain (Fig. 2). The adjacent chains are further interconnected by the *L*^2-^ ligands *via* intermolecular O—H···O hydrogen bonds (Table 1) to form a three-dimensional supramolecular framework (Fig. 3).

### Experimental

10 mL Pyrex glass tube was loaded by NiCl_2_.6H_2_O (24 mg, 0.1 mmol), 3,3'-(1,4-phenylene)-diacrylic acid (22 mg, 0.1 mmol), 4,4'-bipyridyl (16 mg, 0.1 mmol), and 3 ml of H_2_O. The tube was sealed and heated in an oven to 170°C for 3 d, and then cooled to ambient temperature at the rate of 5°C h^-1^ to form blue crystals.

### Refinement

The H atoms of the coordinated water molecules were located on a difference Fourier map and isotropically refined. All the rest H atoms were placed in geometrically idealized positions (C–H = 0.94 Å)  and constrained to ride on their parent atoms with, *U*_iso_(H) = 1.2*U*_eq_(C).

### Crystal data

**Table:**

[Ni(C_10_H_8_N_2_)(H_2_O)_4_](C_12_H_8_O_4_)	*Z* = 1
*M**_r_* = 503.12	*F*(000) = 262
Triclinic, *P*1	*D*_x_ = 1.600 Mg m^−^^3^
Hall symbol: -P 1	Mo *K*α radiation, λ = 0.71073 Å
*a* = 7.0867 (14) Å	Cell parameters from 2063 reflections
*b* = 7.3614 (15) Å	θ = 3.2–25.4°
*c* = 10.418 (2) Å	µ = 0.98 mm^−^^1^
α = 95.51 (3)°	*T* = 223 K
β = 102.51 (3)°	Block, blue
γ = 97.27 (3)°	0.40 × 0.40 × 0.25 mm
*V* = 522.0 (2) Å^3^	

### Data collection

**Table:**

Rigaku Mercury CCD area-detector diffractometer	1884 independent reflections
Radiation source: fine-focus sealed tube	1807 reflections with *I* > 2σ(*I*)
graphite	*R*_int_ = 0.018
ω scans	θ_max_ = 25.4°, θ_min_ = 3.2°
Absorption correction: multi-scan (REQAB; Jacobson, 1998)	*h* = −8→8
*T*_min_ = 0.694, *T*_max_ = 0.791	*k* = −8→7
4910 measured reflections	*l* = −12→12

### Refinement

**Table:**

Refinement on *F*^2^	Primary atom site location: structure-invariant direct methods
Least-squares matrix: full	Secondary atom site location: difference Fourier map
*R*[*F*^2^ > 2σ(*F*^2^)] = 0.026	Hydrogen site location: inferred from neighbouring sites
*wR*(*F*^2^) = 0.067	H atoms treated by a mixture of independent and constrained refinement
*S* = 1.07	*w* = 1/[σ^2^(*F*_o_^2^) + (0.0333*P*)^2^ + 0.2568*P*] where *P* = (*F*_o_^2^ + 2*F*_c_^2^)/3
1884 reflections	(Δ/σ)_max_ < 0.001
167 parameters	Δρ_max_ = 0.41 e Å^−^^3^
0 restraints	Δρ_min_ = −0.36 e Å^−^^3^

### Special details

**Table:**

Geometry. All e.s.d.'s (except the e.s.d. in the dihedral angle between two l.s. planes) are estimated using the full covariance matrix. The cell e.s.d.'s are taken into account individually in the estimation of e.s.d.'s in distances, angles and torsion angles; correlations between e.s.d.'s in cell parameters are only used when they are defined by crystal symmetry. An approximate (isotropic) treatment of cell e.s.d.'s is used for estimating e.s.d.'s involving l.s. planes.
Refinement. Refinement of *F*^2^ against ALL reflections. The weighted *R*-factor *wR* and goodness of fit *S* are based on *F*^2^, conventional *R*-factors *R* are based on *F*, with *F* set to zero for negative *F*^2^. The threshold expression of *F*^2^ > σ(*F*^2^) is used only for calculating *R*-factors(gt) *etc*. and is not relevant to the choice of reflections for refinement. *R*-factors based on *F*^2^ are statistically about twice as large as those based on *F*, and *R*- factors based on ALL data will be even larger.

### Fractional atomic coordinates and isotropic or equivalent isotropic displacement parameters (Å^2^)

**Table:**

	*x*	*y*	*z*	*U*_iso_*/*U*_eq_	
Ni1	0.5000	1.0000	0.0000	0.01822 (12)	
N1	0.6792 (2)	1.0320 (2)	0.19330 (14)	0.0216 (3)	
O1	0.2663 (2)	1.0289 (2)	0.08405 (13)	0.0259 (3)	
H1W	0.209 (4)	1.105 (4)	0.052 (3)	0.044 (8)*	
H2W	0.182 (4)	0.935 (4)	0.075 (3)	0.055 (8)*	
O2	0.4632 (2)	0.72250 (19)	0.01349 (16)	0.0302 (3)	
H3W	0.565 (5)	0.678 (4)	0.048 (3)	0.062 (9)*	
H4W	0.391 (4)	0.643 (4)	−0.042 (3)	0.056 (8)*	
O3	−0.03762 (19)	0.74413 (19)	0.03648 (14)	0.0323 (3)	
O4	−0.22675 (19)	0.58018 (19)	0.14203 (14)	0.0303 (3)	
C1	0.6170 (3)	0.9485 (3)	0.28808 (19)	0.0298 (4)	
H1	0.4846	0.8961	0.2712	0.036*	
C2	0.7352 (3)	0.9347 (3)	0.40847 (19)	0.0297 (4)	
H2	0.6832	0.8755	0.4720	0.036*	
C3	0.9324 (3)	1.0085 (2)	0.43649 (17)	0.0206 (4)	
C4	0.9949 (3)	1.0998 (3)	0.33922 (18)	0.0262 (4)	
H4	1.1259	1.1554	0.3539	0.031*	
C5	0.8665 (3)	1.1094 (3)	0.22164 (18)	0.0249 (4)	
H5	0.9127	1.1735	0.1581	0.030*	
C6	0.3586 (3)	0.5704 (3)	0.5528 (2)	0.0317 (5)	
H6	0.2626	0.6181	0.5898	0.038*	
C7	0.4735 (3)	0.4638 (3)	0.3653 (2)	0.0325 (5)	
H7	0.4564	0.4381	0.2730	0.039*	
C8	0.3284 (3)	0.5354 (3)	0.41562 (19)	0.0266 (4)	
C9	0.1461 (3)	0.5739 (3)	0.3316 (2)	0.0303 (4)	
H9	0.0401	0.5840	0.3713	0.036*	
C10	0.1196 (3)	0.5953 (3)	0.2054 (2)	0.0308 (4)	
H10	0.2231	0.5798	0.1642	0.037*	
C11	−0.0630 (3)	0.6423 (3)	0.12331 (19)	0.0246 (4)	

### Atomic displacement parameters (Å^2^)

**Table:**

	*U*^11^	*U*^22^	*U*^33^	*U*^12^	*U*^13^	*U*^23^
Ni1	0.01467 (17)	0.02099 (18)	0.01691 (18)	0.00316 (12)	−0.00192 (12)	0.00425 (12)
N1	0.0182 (7)	0.0255 (8)	0.0190 (8)	0.0030 (6)	−0.0006 (6)	0.0042 (6)
O1	0.0187 (7)	0.0328 (8)	0.0264 (7)	0.0062 (7)	0.0024 (6)	0.0080 (6)
O2	0.0252 (8)	0.0213 (7)	0.0382 (8)	0.0035 (6)	−0.0057 (6)	0.0043 (6)
O3	0.0262 (7)	0.0367 (8)	0.0355 (8)	0.0076 (6)	0.0036 (6)	0.0165 (7)
O4	0.0220 (7)	0.0323 (7)	0.0343 (8)	0.0025 (6)	0.0005 (6)	0.0086 (6)
C1	0.0180 (9)	0.0406 (12)	0.0268 (10)	−0.0029 (8)	−0.0017 (8)	0.0102 (9)
C2	0.0216 (9)	0.0417 (12)	0.0227 (10)	−0.0039 (8)	0.0000 (8)	0.0125 (9)
C3	0.0198 (9)	0.0205 (9)	0.0185 (9)	0.0022 (7)	−0.0013 (7)	0.0026 (7)
C4	0.0177 (9)	0.0336 (11)	0.0228 (9)	−0.0028 (8)	−0.0023 (7)	0.0064 (8)
C5	0.0228 (9)	0.0298 (10)	0.0197 (9)	−0.0010 (8)	0.0010 (7)	0.0063 (8)
C6	0.0256 (10)	0.0386 (12)	0.0336 (11)	0.0115 (9)	0.0085 (9)	0.0054 (9)
C7	0.0359 (11)	0.0406 (12)	0.0205 (10)	0.0101 (9)	0.0025 (8)	0.0059 (9)
C8	0.0228 (9)	0.0247 (10)	0.0298 (10)	0.0034 (8)	−0.0009 (8)	0.0074 (8)
C9	0.0242 (10)	0.0321 (11)	0.0340 (11)	0.0048 (8)	0.0033 (8)	0.0078 (9)
C10	0.0228 (10)	0.0344 (11)	0.0342 (11)	0.0049 (8)	0.0022 (8)	0.0087 (9)
C11	0.0225 (9)	0.0214 (9)	0.0261 (10)	0.0034 (7)	−0.0018 (8)	0.0011 (8)

### Geometric parameters (Å, °)

**Table:**

Ni1—O2^i^	2.0486 (14)	C2—H2	0.9400
Ni1—O2	2.0487 (14)	C3—C4	1.387 (3)
Ni1—O1^i^	2.0582 (14)	C3—C3^ii^	1.483 (3)
Ni1—O1	2.0582 (14)	C4—C5	1.372 (3)
Ni1—N1^i^	2.1093 (16)	C4—H4	0.9400
Ni1—N1	2.1093 (16)	C5—H5	0.9400
N1—C5	1.334 (2)	C6—C7^iii^	1.373 (3)
N1—C1	1.336 (2)	C6—C8	1.391 (3)
O1—H1W	0.79 (3)	C6—H6	0.9400
O1—H2W	0.84 (3)	C7—C6^iii^	1.373 (3)
O2—H3W	0.85 (3)	C7—C8	1.389 (3)
O2—H4W	0.82 (3)	C7—H7	0.9400
O3—C11	1.257 (2)	C8—C9	1.472 (3)
O4—C11	1.255 (2)	C9—C10	1.314 (3)
C1—C2	1.370 (3)	C9—H9	0.9400
C1—H1	0.9400	C10—C11	1.489 (3)
C2—C3	1.391 (3)	C10—H10	0.9400
			
O2^i^—Ni1—O2	180.0	C3—C2—H2	120.1
O2^i^—Ni1—O1^i^	90.45 (7)	C4—C3—C2	116.20 (16)
O2—Ni1—O1^i^	89.55 (7)	C4—C3—C3^ii^	122.0 (2)
O2^i^—Ni1—O1	89.55 (7)	C2—C3—C3^ii^	121.8 (2)
O2—Ni1—O1	90.45 (7)	C5—C4—C3	120.40 (17)
O1^i^—Ni1—O1	180.0	C5—C4—H4	119.8
O2^i^—Ni1—N1^i^	86.37 (7)	C3—C4—H4	119.8
O2—Ni1—N1^i^	93.63 (7)	N1—C5—C4	123.09 (17)
O1^i^—Ni1—N1^i^	88.07 (6)	N1—C5—H5	118.5
O1—Ni1—N1^i^	91.93 (6)	C4—C5—H5	118.5
O2^i^—Ni1—N1	93.63 (7)	C7^iii^—C6—C8	120.99 (19)
O2—Ni1—N1	86.37 (7)	C7^iii^—C6—H6	119.5
O1^i^—Ni1—N1	91.93 (6)	C8—C6—H6	119.5
O1—Ni1—N1	88.07 (6)	C6^iii^—C7—C8	121.49 (18)
N1^i^—Ni1—N1	180.0	C6^iii^—C7—H7	119.3
C5—N1—C1	116.72 (15)	C8—C7—H7	119.3
C5—N1—Ni1	122.36 (12)	C7—C8—C6	117.51 (18)
C1—N1—Ni1	120.09 (12)	C7—C8—C9	123.34 (18)
Ni1—O1—H1W	109.4 (19)	C6—C8—C9	119.14 (18)
Ni1—O1—H2W	116.4 (19)	C10—C9—C8	125.23 (19)
H1W—O1—H2W	105 (3)	C10—C9—H9	117.4
Ni1—O2—H3W	116 (2)	C8—C9—H9	117.4
Ni1—O2—H4W	125 (2)	C9—C10—C11	124.51 (19)
H3W—O2—H4W	109 (3)	C9—C10—H10	117.7
N1—C1—C2	123.70 (17)	C11—C10—H10	117.7
N1—C1—H1	118.1	O4—C11—O3	124.63 (17)
C2—C1—H1	118.1	O4—C11—C10	120.49 (17)
C1—C2—C3	119.80 (17)	O3—C11—C10	114.88 (17)
C1—C2—H2	120.1		

Symmetry codes: (i) −*x*+1, −*y*+2, −*z*; (ii) −*x*+2, −*y*+2, −*z*+1; (iii) −*x*+1, −*y*+1, −*z*+1.

### Hydrogen-bond geometry (Å, °)

**Table:**

*D*—H···*A*	*D*—H	H···*A*	*D*···*A*	*D*—H···*A*
O1—H2W···O3	0.84 (3)	1.90 (3)	2.734 (2)	170 (3)
O1—H1W···O3^iv^	0.79 (3)	1.90 (3)	2.683 (2)	171 (3)
O2—H3W···O4^v^	0.85 (3)	1.86 (3)	2.701 (2)	172 (3)
O2—H4W···O4^vi^	0.82 (3)	1.95 (3)	2.754 (2)	167 (3)

Symmetry codes: (iv) −*x*, −*y*+2, −*z*; (v) *x*+1, *y*, *z*; (vi) −*x*, −*y*+1, −*z*.
